# Intraoperative molecular imaging clinical trials: a review of 2020 conference proceedings

**DOI:** 10.1117/1.JBO.26.5.050901

**Published:** 2021-05-17

**Authors:** Feredun Azari, Gregory Kennedy, Elizabeth Bernstein, Costas Hadjipanayis, Alexander L. Vahrmeijer, Barbara L. Smith, Eben Rosenthal, Baran Sumer, Jie Tian, Eric R. Henderson, Amy Lee, Quyen Nguyen, Summer L. Gibbs, Brian W. Pogue, Daniel A. Orringer, Patra Charalampaki, Linda W. Martin, Janos L. Tanyi, Major Kenneth Lee, John Y. K. Lee, Sunil Singhal

**Affiliations:** a University of Pennsylvania, Perelman School of Medicine, Philadelphia, Pennsylvania, United States; b Icahn School of Medicine at Mount Sinai, New York, United States; c Leiden University, Medical Center, Leiden, The Netherlands; d Harvard University, School of Medicine, Boston, Massachusetts, United States; e Stanford University, School of Medicine, Stanford, California, United States; f University of Texas Southwestern Medical Center, Dallas, Texas, United States; g Chinese Academy of Sciences/Institute of Automation, Beijing, China; h Dartmouth College, Geisel School of Medicine, Hanover, New Hampshire, United States; i University of Washington, School of Medicine, Seattle, Washington, United States; j University of California San Diego, School of Medicine, San Diego, California, United States; k Oregon Health & Science University, Knight Cancer Institute, School of Medicine, Portland, Oregon, United States; l Thayer School of Engineering at Dartmouth, Hanover, New Hampshire, United States; m University of Michigan, Ann Arbor, Michigan, United States; n Cologne Medical Center, Cologne, Germany; o University of Virginia, School of Medicine, Charlottesville, Virginia, United States

**Keywords:** intraoperative molecular imaging, fluorescence-guided surgery, tumor surgery, optical biopsy, intraoperative visualization, molecular imaging

## Abstract

**Significance:** Surgery is often paramount in the management of many solid organ malignancies because optimal resection is a major factor in disease-specific survival. Cancer surgery has multiple challenges including localizing small lesions, ensuring negative surgical margins around a tumor, adequately staging patients by discriminating positive lymph nodes, and identifying potential synchronous cancers. Intraoperative molecular imaging (IMI) is an emerging potential tool proposed to address these issues. IMI is the process of injecting patients with fluorescent-targeted contrast agents that highlight cancer cells prior to surgery. Over the last 5 to 7 years, enormous progress has been achieved in tracer development, near-infrared camera approvals, and clinical trials. Therefore, a second biennial conference was organized at the University of Pennsylvania to gather surgical oncologists, scientists, and experts to discuss new investigative findings in the field. Our review summarizes the discussions from the conference and highlights findings in various clinical and scientific trials.

**Aim:** Recent advances in IMI were presented, and the importance of each clinical trial for surgical oncology was critically assessed. A major focus was to elaborate on the clinical endpoints that were being utilized in IMI trials to advance the respective surgical subspecialties.

**Approach:** Principal investigators presenting at the Perelman School of Medicine Abramson Cancer Center’s second clinical trials update on IMI were selected to discuss their clinical trials and endpoints.

**Results:** Multiple phase III, II, and I trials were discussed during the conference. Since the approval of 5-ALA for commercial use in neurosurgical malignancies, multiple tracers and devices have been developed to address common challenges faced by cancer surgeons across numerous specialties. Discussants also presented tracers that are being developed for delineation of normal anatomic structures that can serve as an adjunct during surgical procedures.

**Conclusions:** IMI is increasingly being recognized as an improvement to standard oncologic surgical resections and will likely advance the art of cancer surgery in the coming years. The endpoints in each individual surgical subspecialty are varied depending on how IMI helps each specialty solve their clinical challenges.

## 1 Introduction

Malignant neoplasms account for the second leading cause of death worldwide and it is estimated that, by 2040, the incidence of such neoplasms will increase by almost 50%.^1^ Despite advances in noninvasive management of these diseases, surgery remains an integral part of curative treatment and will continue to be an important aspect of the management of solid tumors. To date, the single most important predictor of a successful cancer outcome for almost every solid cancer is a complete resection during surgery. In fact, across the spectrum of cancers, complete resection (R0) of disease has been shown to reduce recurrence and increase disease-free survival (DFS).^2^

However, this surgical goal remains a challenge for oncologic surgeons across specialties because patients frequently present with synchronous or metachronous lesions not detected in preoperative radiographic evaluations, have large tumor burden, abut or invade critical structures in the vicinity, and have residual disease in the tumor bed due to inadequate surgical margins or localized skip metastases.^3^ Conventional means of obtaining a complete resection during surgery have relied on tactile and visual assessment intraoperatively by the surgeon. The rise of minimally invasive techniques has hampered the ability of finger palpation to detect malignant lesions. Additional challenges include adequate and appropriate staging of patients such as identifying lymph nodes containing micrometastatic disease, thereby altering the postoperative management of this subgroup of patients.

Over the last decade, one of the tools that has been key to addressing these challenges has been intraoperative molecular imaging (IMI). IMI utilizes optical tracers that target malignant cells and make them fluoresce intraoperatively. Thus, this technology allows the surgeon to visually detect the fluorescent cancer cells, drawing attention to missed lesions and unexpected nodules and thereby allowing the surgeon to remove them in an oncologically sound manner in real time.^4^^–^^6^

Successful implementation of IMI is predicated upon cancer-specific fluorescent optical tracers. If the tracers are in the nonvisible optical frequency (>∼650  nm), an optical imaging system is requisite to detecting the fluorescence in the target of interest. Developments in these components have propelled IMI over the last two decades and have made it a critical element in various oncologic operations. IMI has also grown in popularity due to its ease of use and interpretation, real-time integration into current surgical protocols, and lack of side effects common to other imaging technologies, including exposure to ionizing radiation.^7^

Notwithstanding the recent advancements of IMI, the technology remains in its infancy. Recently, the first two agents, 5-aminolevulinic acid (5-ALA) and hexaminolevulinate, were approved as IMI tracers by the U.S. Food and Drug Administration (FDA).^8^ However, given their intrinsic limitations, the scientific community is in constant exploration of other tracers and optical imaging devices. A considerable amount of current research focus has centered on developing tracers that are specific for certain tumor targets. However, similar advances have been made in optical camera and computational systems that detect fluorescence intensity with increasing precision. This symbiotic relationship now allows for the detection of smaller lesions deeper in parenchyma utilizing the near-infrared (NIR) I and NIR-II spectrum. Ultimately, this translates to better disease clearance and minimized cancer recurrences, leading to improved patient outcomes.

As IMI continues to present successful results in optimal oncologic resection, researchers look to explore its potential beyond tumor identification. Large morbidity associated with a lot of oncologic resections stems from damage to nearby normal critical structures such as nerves, visceral organs, and vasculature. Therefore, a recent focus of IMI research has been on developing contrast agents and imaging devices that help identify the aforementioned critical structures. Positive developments in this area will help improve patient outcomes by avoiding morbid injuries and rapidly identify injuries at the time of occurrence. Despite the known benefits and applicability of IMI in the current clinical realm, a wider awareness of its potential is still lacking in the surgical fields.

Thus, to explore the developments in the field of IMI, a group of 300 researchers and industry partners from 38 US states and 10 countries came together at the Perelman School of Medicine at the University of Pennsylvania on November 6, 2020. This was the second biennial IMI conference hosted at the University of Pennsylvania. The objective of this conference was to discuss lessons learned across multiple specialties from phase 3, phase 2, and emerging phase 1 IMI clinical trials. The goal of this unique conference was to evaluate the real-time clinical value that IMI provides clinicians and its impact on the outcome of cancer patients across different specialties. This paper summarizes the salient points in the discussions surrounding the advances in IMI over the last decade presented by leaders and innovators in the field (Table 1).

**Table 1:** Summary of tracer trials currently in phase 3 and beyond.

Presenter	Tracer	Tumor type
Costas Hadjipanayis, MD, PhD	5-ALA (Gleolan)	Various neurosurgical applications
Janos L. Tanyi, MD, PhD	FRa	Ovarian cancer
Sunil Singhal, MD	FRa	Lung cancer
Alexander L. Vahrmeijer, MD	CEA (SGM-101)	Colon cancer
Barbara L. Smith, MD, PhD	Protease-activated dye	Breast cancer

## 2 Phase 3/4 Trial

### 2.1 Summary of Important Points

• 5-ALA (Gleolan) is becoming more prevalent among neurosurgeons for management of high-grade gliomas (HGG) since FDA approval.
• Current applications for 5-ALA extend beyond fluorescence-guided surgery and explore its use in theranostics and real-time margin assessments.
• OTL38, which is a folate receptor-alpha-targeted NIR tracer, has allowed for occult lesion detection in 48% of patients with ovarian cancer that would have otherwise been missed using current conventional techniques.
• Phase 3 results of OTL38 in ovarian cancer are expected to be published soon and will build on previous successful findings in earlier trials.
• OTL38 similarly produced encouraging results in surgical management of lung adenocarcinomas.
• Use of IMI via OTL38 has allowed surgeons to localize primary tumors, assess margins, and identify occult synchronous lesions, resulting in 26% improvement in surgical outcomes.
• SGM-101 [anticarcinoembryonic antigen (CEA)-based tracer] is a specific targeted tracer for tumors that express CEA. >90% of colorectal malignancies express CEA, highlighting the broad applicability in management of early and advanced colorectal cancers.
• SGM-101 is currently being studied in a multinational, multi-institutional trial. The tracer is being evaluated for its ability to detect primary, occult, and metastatic lesions in advanced colorectal patients.
• LUM015, a protease-activated tracer, has broad applicability in breast cancer surgery, where it has been found to reduce re-excision rates and detection of positive margins that would have otherwise been missed by conventional techniques.

### 2.2 5-ALA: Postapproval Adoption Challenges, Reimbursement, and Future Directions: Phase IV

Discussion of phase 3 trials and phase 4 data started with Dr. Hadjipanayis and the Mount Sinai experience with 5-ALA for neurosurgical procedures, particularly HGG post FDA approval.^8^ Dr. Hadjipanayis initiated the discussion by summarizing the scarce systemic and surgical treatment options available for HGG patients and how it has evolved over the last four decades. The goal of HGG research and development is to enhance the standard of care surgical resection of HGG, which is an R0 resection. Currently, neurosurgeons focus on contrast enhanced borders on imaging, but Dr. Hadjipanayis demonstrated that HGG often extends beyond these regions and leads to cancer-positive margins.

Positive margins are often invisible to the naked eye and have been the Achilles heel of HGG. However, 5-ALA, which is taken orally presurgery, undergoes a biosynthetic reaction into an active metabolite protoporphyrin IX, which is excited at 405 nm and can be seen with blue-violet illumination 635-nm intraoperatively. Several studies were then reviewed; they demonstrated a high positive predictive value (PPV) (97%) and diagnostic accuracy of >90% for 5-ALA in HGG.^9^ One of the studies, of which Mount Sinai was a participant in a multicenter investigation, preliminarily showed a 99% PPV for detection of HGG. Overall, these results paved the way for 5-ALA approval by the FDA.^8^^–^^10^

The focus of the discussion then turned toward the postapproval experience of Gleolan (NX Development Corporation, Lexington, Kentucky) and the challenges faced by the HGG community. To date, 621 neurosurgeons in the United States have been trained to use Gleolan with 177 centers adding the tracer into their formularies. Interestingly, despite the recent approval, almost 50% of the HGG cases today in the United States use Gleolan, with the number expected to grow. Worldwide, 80,000 cases of HCG resections have been done with the aid of Gleolan. Widespread acceptance of this agent has resulted in the addition of a unique ICD-10 code for reimbursement and increased insurance coverage through major insurers. However, the technology is not without its challenges, which include resistance to the adoption of IMI as part of surgical care, the need for new operative microscopes for visualization, the cost of the tracer, and the need for administering Gleolan several hours preoperatively.^8^^,^^11^

Dr. Hajipanayis concluded by discussing the future directions of 5-ALA. Current investigations include looking at the timing of 5-ALA administration prior to surgery, with data pointing toward better visualization with an increased interval from tracer ingestion. Some of the technological advancements that further enhance 5-ALA were also discussed, including new exoscopes in the operating room, handheld fluorescence imaging, pushing the boundaries of diagnostic imaging, and the potential role of 5-ALA in theranostics. Dupont et al. showed that the combination of 5-ALA and PDT provides better disease control in the surgical cavity. In conclusion, since the approval of 5-ALA, IMI has made significant strides and continues to expand beyond HGG management.^12^

### 2.3 Folate NIR Tracer for Ovarian Cancer

Dr. Janos Tanyi from the University of Pennsylvania Perelman School of Medicine discussed the current phase 3 trial of the folate receptor alpha (FRa) tracer OTL38 (On Target Laboratories, West Lafayette, Indiana) for ovarian cancer. This is an NIR tracer that links a fluorochrome to folate and selectively binds the folate receptor. The fluorochrome has an excitation wavelength of 774 nm and emission wavelength of 794 nm. Dr. Tanyi discussed the outcome improvements in patients with advanced ovarian cancer who undergo cytoreductive surgery. Ovarian cancer typically ruptures and seeds the peritoneal cavity, thus, cytoreductive surgery is considered an important means of disease control. Dr. Tanyi highlighted multiple studies that have shown improved DFS when residual disease is decreased down to <1  cm. However, this is often difficult to achieve with standard-of-care surgery as there are multiple tumor deposits that are not identified with visual inspection. This challenge can be reduced by the innovation of IMI, particularly by folate-targeted tracers, as the majority of ovarian cancers express FRa even after neoadjuvant therapy.

Dr. Tanyi reviewed his prior experience with OTL38 in a single-arm, open label, multicenter phase 2 trial involving 48 subjects.^13^ The primary endpoints were to assess the sensitivity and PPV of OTL38 to detect FRa-positive ovarian cancer. A secondary endpoint of the study was to assess the safety of OTL38 in the cohort. With regard to safety, fewer than 18% of patients experienced an adverse drug-related event. This is consistent with prior pilot studies of OTL38. In terms of clinical utility, 48% of the subjects had additional lesions that were missed by visual inspection but were detected using OTL38-based imaging.^13^ Favorable results from this study have propelled the ongoing phase 3 study of OTL38 in ovarian malignancies.

The primary goal of the current multi-institutional, randomized phase 3 trial is to confirm the efficacy of OTL38 in combination with fluorescent light to detect additional folate receptor-positive ovarian cancer lesions not detected by palpation and visualization under normal light in patients with FRa-positive ovarian cancers scheduled to undergo primary surgical cytoreduction, interval debulking, or recurrent ovarian cancer surgery. Secondary endpoints include the estimation of the proportion of FR-positive ovarian cancer patients in whom all lesions, without regard to evaluable lesion status, are detected by fluorescent light and its concordance with final pathological evaluation for receptor status. In addition, investigators will further explore the safety of the infusion drug in the study cohort.

In the phase 3 study, patients receive a single dose of 0.025  mg/kg of OTL38 at least 1 h before surgery. Then during the surgery, white-light assessment is performed to identify tumor nodules. Prior to any resection, the patient is then randomized in the operating room to surgery using white-light only versus white-light and NIR. Those randomized to the white-light only group would undergo current standard of care cytoreductive surgery based on visual and tactile feedback. This is a multi-institutional study involved 10 centers with ∼178 subjects enrolled. Study results are expected to be published soon after the final analysis of results. This study has neared completion. These results in conjunction with prior reports in the literature are expected to advance real-life clinical improvements in ovarian cancer patients undergoing cytoreductive surgery.

### 2.4 Folate NIR Dye for Lung Cancer

Dr. Sunil Singhal from the Perelman School of Medicine at the University of Pennsylvania discussed ongoing applications of FR-targeted imaging using OTL38 for lung adenocarcinomas. Dr. Singhal pointed out that IMI has the potential to address three intraoperative challenges in lung malignancy resections. These include the ability to localize primary tumors, assess margins, and identify occult synchronous lesions not detected on the preoperative evaluation.^14^

**Fig. 1:**
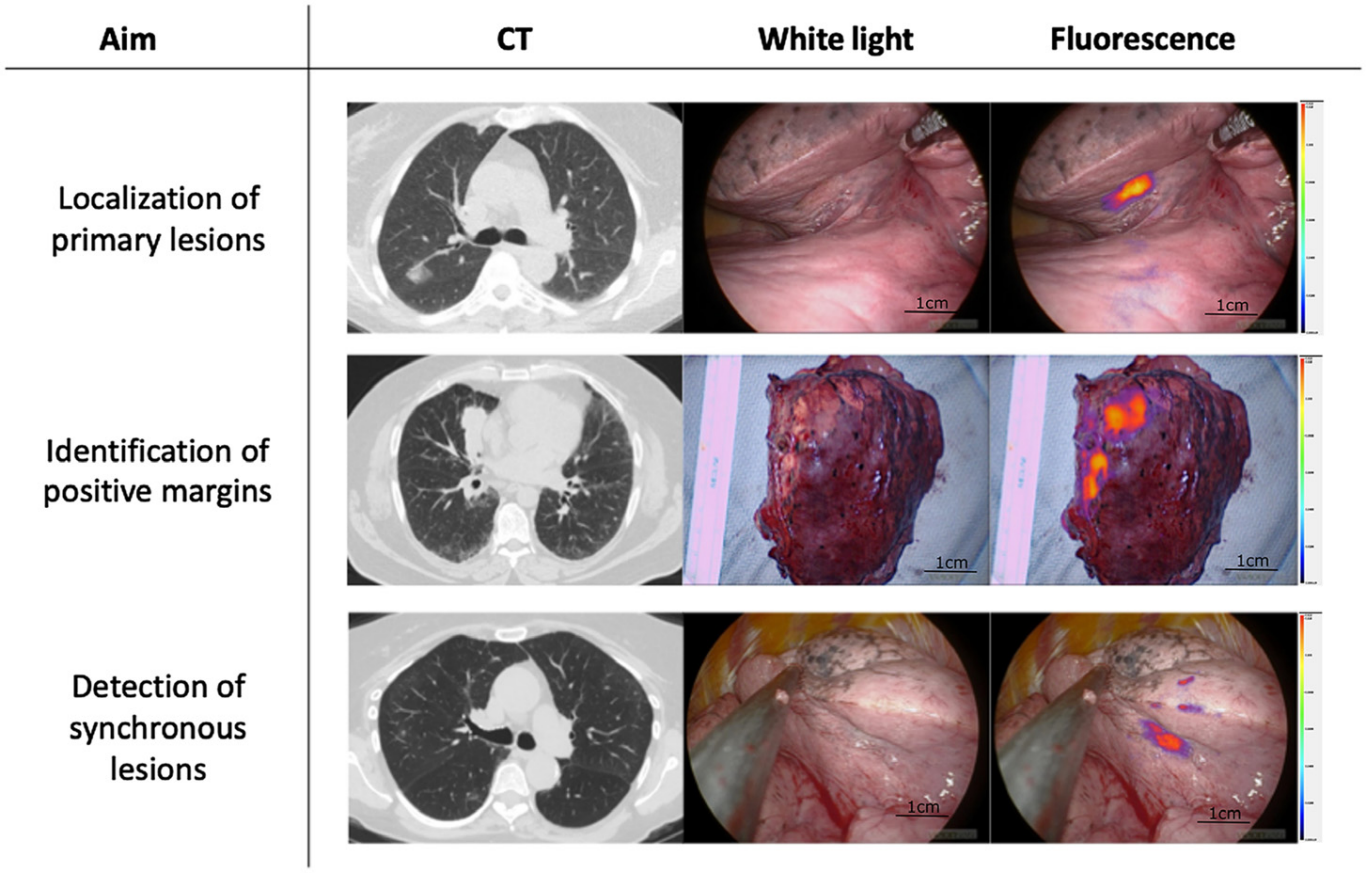
OTL38 use in lung adenocarcinoma not only aids in identifying primary lesions but also has shown utility in detecting positive surgical margins on resected specimens and detecting synchronous lesions that were missed on preoperative evaluation.

The benefits of FR-targeted IMI have been previously demonstrated by early pilot and phase 1 studies. Predina et al.^15^ showed that OTL38 can detect subcentimeter solid lung malignancies as well as ground glass opacities, which are thought to be precursors to cancerous lesions. In addition, during those studies involving 30 patients, four had lesions detected only by IMI and were missed using standard surgical methods. Given these favorable preliminary results, a phase 2 multicenter study was undertaken, with the primary goals of identifying occult synchronous lesions, localizing of primary tumors, and identifying positive margins. 110 subjects were enrolled in the study with 92 patients included in the final analysis. Results from the study were encouraging, as there were nine patients with synchronous lesions detected using OTL38, 11 tumors localized using IMI that were missed with standard methods, and nine positive margins detected in real time prior to pathological evaluation. Together, there was almost a 26% improvement in surgical outcomes by adding IMI to the standard-of-care surgical resections. Overall, similar to previous reports, there were minimal drug-related toxicities noted in 12 patients.^16^ Representative images of OTL38 use in lung adenocarcinoma are shown in Fig. 1.

These and previous reports have demonstrated the safety and efficacy of OTL38 and IMI in thoracic oncology as this technology improves identification of occult tumors, facilitates localization of primary tumors, and improves real-time intraoperative margin assessment. After elaborating upon this encouraging data, Dr. Singhal then discussed the multicenter randomized phase 3 trial evaluating OTL38 for lung malignancies. Accrual is currently ongoing, and results are expected to be published over the next 12 to 15 months.

### 2.5 (SGM-101) CEA Colon Cancer Trial

Dr. Vahrmeijer from Leiden University Medical Center in the Netherlands presented their experience with the CEA Colon Cancer Trial. Dr. Vahrmeijer started his discussion with background on the IMI tracer that is used in the trial. SGM-101 (Surgimab, Montpellier, France) is an anti-CEA receptor-targeted antibody coupled with a BM-104 tracer with an emission wavelength of 700 nm. CEA was chosen due to overexpression of this glycoprotein in more than 95% of digestive tumors, and its expression is not typically lost following neoadjuvant therapy.^17^

Preliminary studies for SGM-101 have been encouraging. In a phase 2 trial of 37 patients with biopsy-proven colorectal cancer, the tracer localized the lesion in 27 patients (75%) with a mean tumor-to-background ratio of 1.7. Seven patients had complete pathological response after neoadjuvant therapy, and two patients had false-positive results.^18^ There were no false-negative results in this study. Clinically significant events were noted in six patients in whom eight additional lesions were identified, and nine patients had their surgical plans altered due to IMI findings.^19^ In another study, SGM-101 successfully identified distant CEA-expressing metastases including liver and lymph nodes for those diagnosed with colorectal as well as pancreatic adenocarcinomas. In addition, the utility of SGM-101 was explored in HIPEC for extensive colorectal diseases. The tracer showed 98.5% sensitivity and 62.2% specificity. Overall, in a 14-patient study, PPV was 82.3% and negative predictive value (NPV) was 95.8%.^20^

Dr. Vahrmeijer then focused his discussion on the ongoing phase 3 study of SGM-101. This is a multinational, multi-intuitional study that includes centers from Italy, Germany, the Netherlands, and the United States. The contrast agent will be used to localize primary and recurrent colorectal tumors as well as to detect infiltrated lymph nodes, liver metastases, and peritoneal metastases. The objectives of the study will be to determine the clinical benefit in achieving R0 resection, to alter the surgical plan based on the findings, and to perform additional resections based on fluorescence noted during surgery. The study population will consist of those diagnosed with T4 colon cancer, T3/4 rectal cancer and recurrent colon cancer and those with peritoneal dissemination. Given the earlier pilot studies, patients will be administered 10 mg of the tracer 3 to 5 days preoperatively. The goal is to enroll a total of 300 patients, with 96 patients already recruited at the time of this writing. All lesions will be compared with the gold standard surgical technique and histopathologic diagnosis. IMI appears to be an integral part of surgical management of gastrointestinal malignancies in the near future as more studies come to fruition.

### 2.6 Protease-Activated Dye in Breast Cancer Surgery

Dr. Barbara Smith from Harvard Medical School presented two ongoing phase 3 trials in breast surgery. The role of IMI in breast cancer management could be paramount in the future due to the high incidence of the disease, the correlation of R0 resection with overall survival, and the higher incidence of residual disease resection (20% re-excision rate) compared with other specialties owing to intrinsic constraints of native breast tissue.^21^

With these challenges in mind, Dr. Smith’s team has been investigating the clinical utility of Lumicell Imaging Systems in various clinical trials. The imaging system comprises a fluorescent tracer LUM015 (Lumicell.inc, Newton, Massachusetts) that is a cathepsin and MMP-activated fluorophore that is injected intravenously between 2 and 6 h prior to surgery, a handheld imaging device that detects the tracer at a 670 nm wavelength, and proprietary software that analyzes the surgical cavity for residual disease. Dr. Smith noted that the current iteration of the system allows one to scan a 2.6-cm-diameter cavity in under 1 s. Initial feasibility studies conducted in 45 patients by Dr. Smith’s team showed favorable results, with sensitivity of 84% and specificity of 73% and an average tumor-to-background ratio (TBR) of 3.52 to 5.69.^22^ Interestingly, LUM015 fluorescence was not altered by tissue histology, did not show a difference in fluorescence in pre- and postmenopausal subjects, and did not affect the tumor hormonal receptor measurements by the pathologist.^23^

Building on the initial data, investigators then embarked on a multicenter study to further evaluate the efficacy of LUM015. 234 patients across 16 sites were enrolled in the study. The surgeons at participating sites who were not familiar with the imaging system underwent three to four training sessions. Preliminary results from the study confirmed the clinical utility of IMI systems in breast cancer margin assessment. Notably, LUM015 detected residual disease in 11% of subjects after standard of care surgery and located residual cells in 7% of patients that were missed on histopathological evaluation. On final assessment, LUM015 detected positive margins in all of those that were declared positive by the gold standard pathology and detected positive margins in 16% of patients that were missed by the surgeon and pathologist. Overall, the NPV of the system was 97% [sensitivity (SN) 67% and specificity (SP) 70%].^22^

Based on the previous data, Dr. Smith’s team has embarked on two multicenter phase 3 studies. The pivotal study involves 15 sites with primary endpoints of assessing LUM015 on residual tumor excision, assessing sensitivity and specificity, and reducing positive margins. The second study involves five to six sites with the primary endpoints of developing separate algorithms based on tissue histology, reducing residual disease in the tumor bed, and assessing the superiority of the system in evaluating positive margins across tumor subtypes. Currently, both trials are undergoing patient accrual.

## 3 Phase 2 Studies

After the discussion of ongoing phase 3 studies in IMI, attention was turned toward current phase 2 studies. The majority of the presentations built on applications of previously published pilot studies. The topics in this field included IMI complementing pathological assessment, improving surgical management for metastatic disease, new IMI tracers that go beyond simple fluorescence principles, and future directions resulting from these. Topics for the phase 2 section are summarized below (Table 2).

**Table 2:** Summary of tracer trials currently in phase 2 and beyond.

Presenter	Tracer	Tumor type
Eben Rosenthal, MD	Anti-EGFR antibody	Head and neck malignancies
Linda W. Martin, MD, MPH	TumorGlow	Lung cancer, soft tissue sarcomas
Baran Sumer, MD, FACS	pH-sensitive tracers	Prostate cancer

### 3.1 Summary of Important Points

• Panitumumab [anti-epidermal growth factor receptor (EGFR) antibody]-conjugated IRDye800 has broad applicability in various malignancies both *in vitro* and in clinical models.
• Anti-EGFR-based NIR tracers have been validated in head and neck malignancies for detecting additional disease, evaluating sentinel margin negative status, evaluating deep residual margin disease, and orienting sentinel margin positivity.
• TumorGlow [high dose indocyanine green (ICG) infused 24-h prior to surgery] can detect occult pulmonary metastatic sarcoma lesions at a much higher rate compared with conventional techniques, particularly during minimally invasive surgery. Success of TumorGlow appears to be limited to lesions within 2 cm of pleural surface.
• ONM-100 is a pH-activated dye that undergoes conformational change and fluoresce in the slightly acidic tumor microenvironment. Early phase 2 results of ONM-100 for prostate cancer using the Intuitive daVinci Robotic system has been encouraging, and more trials are planned to evaluate its efficacy for other types of malignancies.

### 3.2 Anti-EGFR Antibody Imaging Strategies for Real-Time Surgical and Pathologic Guidance

Dr. Eben Rosenthal from Stanford University School of Medicine discussed clinical studies evaluating the use of fluorescently labeled anti-EGFR antibodies in the detection of primary tumor and lymph nodes in head and neck cancer. Dr. Rosenthal’s group has validated that the tracers bind directly to tumor cells and tumor fluorescence is directly correlated to EGFR expression. Currently, his group is focusing efforts on the use of panitumumab conjugated to IRDye800 for NIR surgical navigation.^24^

It is worth noting some of the advantages of antibody-based surgical imaging. Clinical trials have been conducted in multiple cancer types including brain, colon, and pancreatic malignancies.^24^^–^^27^ Unlike activated probes, antibodies are more likely to be retained within the tumor cell, are less complex and less costly to manufacture, and generate more safety concerns (Table 3).

**Table 3:** Advantages of anti-EGFR antibodies in head and neck cancer.

Advantages of anti-EGFR antibodies in head and neck cancer
1. Have been shown by us to detect very small (<8 mg) pieces of tumor
2. Accumulate rapidly inside the tumor since receptor expression and turnover is higher in tumors than normal tissues (the antibody that binds to EGFR is internalized, and then the receptor recycles to the surface).^26^
3. Are simple to manufacture, highly specific for cancer, and shown to be safe.^27^^,^^28^
4. Can be labeled and successfully target tumors for optical and nuclear imaging.

He then presented four distinct applications of NIR imaging in head and neck cancer, which are applicable for any probe and include detecting additional disease, evaluating sentinel margin negative status, evaluating deep residual margin disease, and orienting sentinel margin positivity.

IMI has an important role in head and neck cancer. Dr. Rosenthal’s clinical studies have demonstrated that, if using a sufficiently specific probe, IMI in head and neck cancer can detect satellite lesions in the oral cavity that were undetectable by white-light and would have otherwise been left behind, resulting in recurrent disease. A positive margin in head and neck cancer is when a tumor approaches within 5 mm of the cut surface of the specimen. Therefore, it is necessary to detect a tumor that is close to the surface of the specimen. IMI can identify and orient the sentinel margin positive status (margin that is most likely to be positive) for the pathologist, as head and neck cancers are notoriously difficult to spatially orient when resected. In addition, lack of fluorescence in an EGFR-positive tumor can reassure the surgeon that the deep margin and sentinel margin negative (margin that is likely to be negative) is truly negative with an appropriate margin distance.^25^ In addition to real-time guidance, IMI can assist the surgeon during analysis of the specimen. After removal, the specimen can be placed inside a closed-field imaging device (LI-COR Biosciences, Lincoln, Nebraska) that allows for quantitative imaging. The relative intensity was then analyzed, and it was found that the closest margin could be identified as the highest intensity area of fluorescence (the sentinel margin).^26^

There is also the potential to use antibody-based IMI to assess tumor-positive and sentinel lymph nodes after systemic administration of the labeled antibody. Compared with conventional means of sentinel node identification in which the gamma tracer is injected directly into tumor tissue, anti-EGFR-labeled tracers are injected systemically. This allows for identification of not only malignant tissues but also any metastatic node, even if it is not in the first draining basin. Dr. Rosenthal concluded his remarks by pointing out that the tracers are not only limited to head and neck malignancies but also have shown efficacy in HGG and pancreatic malignancies.^13^

### 3.3 Alliance Trial: TumorGlow^®^ in Sarcoma Metastasis to Lung

Dr. Linda Martin from the University of Virginia School of Medicine discussed plans for a clinical trial in sarcoma metastasectomy. Dr. Martin pointed out that pulmonary spread of sarcomas is a well-recognized paradigm, but unlike other advanced stage disease, resection of these lesions can improve DFS of patients. Therefore, she explored the use of NIR IMI technology for improved detection of lesions missed by preoperative imaging and traditional intraoperative inspection. The IMI tracer of choice in this setting is TumorGlow (high dose ICG given the day prior to surgery), which targets malignant lesions by the enhanced permeability and retention (EPR) effect. The mechanism of EPR relies on tumors often having leaky/disorganized capillaries that systemically injected tracers can target. The dye fluoresces around 796 nm range (NIR).^28^

Dr. Martin then described the results from the initial TumorGlow studies involving 30 sarcoma patients. In the study cohort, 53 lesions were identified on preoperative imaging. Patients then underwent open resection via thoracotomy or minimally invasive resection. Initially, surgeons used standard of care (palpation, visual inspection) to detect additional lesions and discovered 11 additional lesions in both groups. Subjects then underwent NIR imaging inspection where IMI detected 24 additional lesions, with the minimally invasive group benefitting from enhanced detection (21/24). There were no differences noted in fluorescence for different types of sarcomas (bony versus soft tissue). However, Dr. Martin did note several limitations observed for TumorGlow in sarcomas with depth of penetration (<2  cm from pleural surface) being the primary constraint. TumorGlow has been shown to be safe in this cohort, with no toxicity noted in the study. However, it should be noted that tumor cellularity was directly proportional to fluorescence and inversely proportional to tracer dosing required.^29^

In summary, TumorGlow and IMI identified additional lesions for pulmonary sarcomatous metastasis, particularly during minimally invasive surgery. This serves as an important diagnostic value that can help medical oncologist in deciding whether additional therapies are needed due to the presence of unexpected cancer metastases. Further large multi-institutional studies are being planned to further identify group of patients that can derive benefit from this technology.

### 3.4 pH-Sensitive Dyes

Dr. Sumer from the University of Texas Southwestern Medical Center presented findings on new IMI tracers. Given the lack of sophisticated metabolic machinery within tumors, the tumor microenvironment relies upon inefficient anaerobic metabolism, which generates acidic byproducts. This in turn causes the tumor microenvironment to be slightly acidic (pH 6.8) compared with normal parenchyma (pH 7.4). Dr. Sumer’s team thought to utilize this tumor characteristic when designing a tracer, where the dye would fluoresce after undergoing conformational change secondary to pH changes. As a proof of concept, Dr. Sumer’s team generated a library of 10 nanoprobes, each encoded with a unique fluorophore. The nanoprobes cover the entire physiologic range of pH (4 to 7.4) with 0.3 pH increments. Each nanoprobe maintained a sharp pH transition (on/off <0.25  pH) and a high fluorescence activation ratio (>50-fold between on and off states). This provides a useful toolkit for studying pH regulation in many pathophysiological indications (e.g., cancer and lysosome catabolism) as well as establishing tumor-activatable systems for cancer imaging and drug delivery.^30^

*In vitro* studies previously have shown successful conformation change of nanoparticles with changes in pH, but their utility in surgical resection was not compatible. Dr. Sumer’s team was able to overcome this by conjugating these particles to an ICG fluorophore. Various feasibility studies were then performed, and the investigators were able to identify lesions in different malignant tissues. This has led to initiation of current phase 2 trial of this pH-sensitive tracer ONM-100 (Onconanomedicine, Fort Worth, Texas) to investigate the dosing, safety, and feasibility in various malignancies.^31^

Dr. Sumer presented preliminary data on ONM-100 use in prostate cancer. Subjects in the study were dosed at 1  mg/kg 24 h (±8  h) prior to resection. Malignant tissues were then imaged using the Intuitive daVinci Robotic System with the Firefly endoscope. Results of the study indicated that ONM-100 was successful in identifying the location of primary tumors in prostate cancer using a readily available minimally invasive robotic surgery system. Same day dosing produced similar fluorescent signals compared with those who received it a day before. In addition, IMI in this setting identified lesions that were missed by conventional means. There were no adverse reactions across different dosing schedules. Further randomized studies are in the pipeline to study the prospect of ONM-100 in other malignancies.^31^ Currently, Dr. Sumer’s team has identified more than 10 probes, but they acknowledge tumor acidosis.

## 4 Phase 1 and Pilot Studies

Following a morning of interesting discussions on current ongoing clinical trials, the focus of the conference turned toward pilot studies in the field of IMI. The discussants presented interesting ideas and data on how IMI can push the envelope in surgical oncology across multiple subspecialties. Table 4 summarizes the phase 1 presentations at the conference.

**Table 4:** Summary of phase 1 studies presented at the conference.

Presenter	Tracer	Specialty
Jie Tian, PhD	ICG	Hepatopancreaticobiliary
John Y.K. Lee MD, MSCE	TumorGlow	Neurosurgery
Major Kenneth Lee, IV, MD, PhD	TumorGlow	Hepatopancreaticobiliary
Eric R. Henderson, MD	EGFR	Orthopedic oncology
Amy Lee, MD	Tumor paint	Pediatric neurosurgery
Quyen Nguyen, MD, PhD	Nerve-specific tracers	Head and neck surgery
Summer L. Gibbs, PhD	Oxazine	Various applications

### 4.1 Summary of Important Points

• Utilization of ICG in the NIR-II window (1000 to 1700 nm) with dedicated imaging systems produced less scattering and allowed for lesions detection at greater depth and resolution in a landmark study for hepatocellular carcinomas.
• TumorGlow Second Window ICG has a 94.5% sensitivity and >95% PPV in detection of various brain tumors including HGG.
• TumorGlow is highly effective in evaluating primary pancreatic malignancies. NPV approached nearly 100% in margin assessment during surgical resection.
• ABY-029 is an anti-EGFR affibody (<5% mass of antibody) linked to IRDye 800cw that has been explored in soft tissue sarcomas as 70% of these lesions exhibit EGFR expression. ABY-029 labeled well the viable portions of the imaged tumors in pilot studies and is being explored in early clinical trials.
• TumorPaint (BLZ-100) is a peptide-linked fluorescent tracer based on chlorotoxin used for pediatric brain tumors. In subjects who received BLZ-100 within the optimal imaging dose range, tumors were positive for fluorescence in 80% of cases on a wide range of histologic subtypes. On *ex vivo* analysis, the sensitivity of the tracer was 82% (>95% if specimens from negative cases were excluded) and specificity was 89%.
• ALM-488 is a peptide-linked tracer that is currently being explored in identification of normal nerve structures during head and neck surgeries.
• Oxazine-derived tracers can accurately identify normal neural structures in various surgical settings both in 700- and 800-nm wavelengths.

### 4.2 NIR II: with ICG in Human Liver Tumors

Phase I discussions started with Professor Jie Tian from Chinese Academy of Sciences presenting on the first in-human study of NIR-II imaging for hepatic malignancies. Dr. Tian prefaced his presentation by pointing out that liver malignancies have a more than 70% recurrence rate. Therefore, there is an urgent need for promising interventions to help this patient population. One of the emerging technologies that pushes the boundaries of current IMI technology is the exploration of the NIR II region, which operates in the 1000 to 1700 nm wavelength range. It is thought that this region of optical imaging will produce less scattering, allowing for lesion detection at greater depth and resolution.^31^

Dr. Jian’s team explored an application of NIR-II imaging in their landmark study, which was recently published in *Nature Biomedical Engineering*.^32^ In the pilot study, 23 patients with preoperative stage liver cancer underwent infusion of an ICG tracer specifically designed for this imaging system. In all of the subjects included in the analysis, the NIR-II showed higher TBR compared with traditional fluorescence imaging. In addition, NIR-II improved HCC detection in the resection bed that was otherwise missed by the naked eye and traditional IMI. In subgroup analysis, researchers also observed that NIR-II imaging showed high accuracy in visualizing the differentiation of the HCC. NIR-II imaging remains a promising aspect of future precision guided surgery.^32^

### 4.3 TumorGlow™ Second Window ICG in Brain Tumors

Dr. John Lee from the Perelman School of Medicine at the University of Pennsylvania continued the discussion of current pilot studies for ICG, focusing on surgical applications for glioblastoma management. 5-ALA has been the gold standard in neurosurgical procedures, but it operates at a lower light wavelength, which is marred with various optical contaminants. Therefore, Dr. Lee’s team explored NIR dyes, which allowed for clearer visualization and 30 times increased fluorescence than 5-ALA.^13^

Patients enrolled in this pilot study received TumorGlow™ (5  mg/kg ICG at 24 h prior to resection). In 80 subjects enrolled in this study, the mean TBR was 5.83 with strong early localization of tumors. There were minimal adverse effects related to tracer infusion as well as minimal disruption to normal operative flow. Sensitivity in 137 lesions was 94.5% with a >95% PPV. In addition, this application allowed for tumor visualization through the dura, which is difficult with conventional techniques.^32^

Further analysis by Dr. Lee’s team showed that this approach was beneficial in detecting residual lesions in the tumor bed, which correlated >90% with postoperative gadolinium-enhanced MRI (the gold standard) and survival benefit in those who had these margins resected. In summary, Second Window ICG demonstrated PPV similar to 5-ALA, better NPV compared with 5-ALA, and excellent localization through the dura and normal cortex. Dr. Lee’s team explored ICG in various other tumors including metastatic lesions with excellent results. Many studies are currently in the pipeline to further delineate the application of this approach.^13^^,^^32^

### 4.4 TumorGlow^®^ in Pancreatic Cancer

Dr. Major Kenneth Lee from the University of Pennsylvania continued the conversation on his experience in using this tracer for pancreatic malignancies. One of the important reasons for exploring IMI use in pancreatic malignancies is the high mortality associated with this disease secondary to margin positivity (local recurrence) and occult metastatic disease not discovered during surgical exploration (distant recurrence). These challenges are well suited to being addressed by IMI.

In the initial feasibility trial, patients with resectable pancreatic cancer received 5  mg/kg of ICG 24 h prior to surgery. Patients underwent staging laparoscopy followed by laparoscopic or open pancreatectomy. NIR imaging was performed during staging laparoscopy, after pancreas mobilization, after tumor resection, and on back table (*ex vivo*). Initial results indicated that in invasive malignancies (n=12) all viable tumors fluoresced with mean TBR of 4.69. In addition, fluorescence was also detected in premalignant or low-grade malignant tumors. With respect to margin assessment, in 12/13 cases, fluorescence at margin correlated with pathology. Lack of fluorescence predicted negative margins 100% of the time. In subgroup analysis, Dr. Lee noted that fluorescence may help to identify infiltrative tumors that were otherwise missed on preoperative imaging. In addition, in patients who underwent neoadjuvant chemoradiotherapy, fluorescence correlated with response to therapy, which is often difficult to discern on conventional imaging.^33^

### 4.5 EGFR Affibody: Design and Implementation of Targeted Tumor Dye

Dr. Henderson from Geisel School of Medicine at Dartmouth presented his discussion on EGFR-based affibody tracers in orthopedic oncology, particularly in the management of soft-tissue sarcomas. Dr. Henderson emphasized the importance of successful tumor removal with negative margin for these patients, but this is often challenging due to often invisible microscopic tumor extension. The gold standard R0, wide local excision with negative margins, requires that the tumor be removed with an envelope of normal tissue encompassing the tumor and its surroundings. Current IMI tracers and applications have fallen short in this realm as their intrinsic ability is limited to directly visualizing the tumor, which is unhelpful for cancers requiring radical or wide local excisions, such as sarcomas.

ABY-029 is an anti-EGFR affibody (<5% mass of antibody) linked to IRDye 800cw. Approximately 60% to 70% of soft-tissue sarcomas overexpress EGFR. Preclinical studies demonstrated that ABY-029 dosing 4 to 8 h before tumor dissection optimizes tumor-to-background contrast^34^ and facilitates same-day tracer dosing, a favorable development compared with conventional EGFR-targeted antibody-based tracers that require infusion multiple days before surgery. Following their preclinical work, Dr. Henderson’s team completed a phase 0 microdose trial of ABY-029, evaluating tracer-associated contrast compared with the surrounding, nontumoral tissues.^34^^–^^36^ Preliminary data indicate that overall tumor contrast-to-normal tissue is 3.25. Fluorescence contrast was highest at the tumors’ periphery, with a TBR of 4.25 with a signal-to-noise ratio of 7.43. ABY-029 labeled well the viable portions of the imaged tumors; however, contrast in necrotic tumor regions was low. In an effort to increase whole-tumor contrast, the team has tested in rodents a paired-agent fluorescence imaging strategy with ABY-029 and ICG with positive results. Further rodent work demonstrated that fluorescence in these patients was not affected by preoperative neoadjuvant therapy.

### 4.6 TumorPaint-Imaging Pediatric Brain Tumors

Dr. Amy Lee from Seattle Children’s Hospital presented her findings on IMI in pediatric brain tumors. Brain tumors are the leading cause of death in children, and completeness of resection is the single best predictor of survival for this group. IMI has been postulated to improve the recurrence rate and margin negativity in this population. Dr. Lee’s team has been focusing on the use of TumorPaint (BLZ-100) (Blaze Bioscience, Seattle, Washington) as an IMI tracer of choice for pediatric brain tumors. This is a peptide-linked fluorescent tracer based on chlorotoxin. Overall, it is a well-tolerated drug based on three adult and one pediatric phase 1 studies (n=95).^13^^,^^37^

A dose escalation study performed on 29 patients showed that the optimal dose for imaging ranged from 13.9 to $17.3\;\mathrm{mg}/m^{2}$, with $15\;\mathrm{mg}/m^{2}$ being the dose chosen for future studies. There was no drug-related serious adverse events. In subjects who received BLZ-100 within the optimal imaging dose range, tumors were positive for fluorescence in 18/23 cases (80%) on a wide range of histologic subtypes. On *ex vivo* analysis, the sensitivity of the tracer was 82% (>95% if specimens from negative cases were excluded) and specificity was 89%.^34^

Based on these encouraging results, a randomized blinded study (BB-006) evaluating fluorescence detection of BLZ-100 was initiated. The accrual goal is 114 patients, and enrollment is currently underway. The primary objective of the study is to collect data on SN and SP of BLZ-100 using the Canvas NIR imaging system. In addition, data will be used to evaluate efficacy of the imaging system and tracers in intraoperative detection and visualization of the tumor. The results of this study can pave the way for the first IMI tracer to be approved in the pediatric population.

### 4.7 ALM-488 for Nerve Identification

Dr. Quyen Nguyen from the University of California San Diego and Dr. Summer Gibbs from the Oregon Health & Science University Knight Cancer Institute presented their talk on nerve-specific dyes for normal tissue identification. Iatrogenic nerve injuries are a major source of morbidity in a variety of surgical procedures owing to the difficulty of identifying small nerves intraoperatively. To address this challenge, Dr. Nguyen explored ALM-488 (peptide-linked tracer), which fluorescently labeled both intact and degenerated nerves. Currently, a multi-institutional phase 1 trial is accruing patients.

### 4.8 Nerve-Specific Dye for Intraoperative Nerve Identification

Dr. Summer Gibbs continued the conversation by presenting her work on synthesizing oxazine derivatives for nerve identification in the NIR range. The initial work of her research group focused on derivatives that gave appropriate contrast of neural tissues compared with normal soft tissue. They identified a signal-to-background ratio of 1.75 or above to be the optimal target.^35^ Interestingly, the group of tracers in question was able to identify not only clearly visible nerves but also the buried nerves, which further facilitated safe dissection of these structures in hostile surgical environments. Dr. Gibbs’s team was also able to synthesize additional oxazine derivatives that fluoresced in the 800 nm range and were able to be detected by the current robotic surgical imaging systems. Currently, the group is focusing on developing water-soluble nerve-specific fluorescent probes that offer optimal imaging contrast with minimal toxicity.^36^

## 5 Intraoperative Imaging and Back Table Imaging Platforms

The final academic session of the day transitioned to focus on the technology used to visualize many of the tracers discussed during earlier sessions. The first presentation provided audience members with an overview of the current state of fluorescence imaging systems, and the remaining two presentations covered the use of imaging platforms in neurosurgery. Session 4 presentation topics are summarized in Table 5.

**Table 5:** Summary of imaging platform studies presented at the conference.

Presenter	Imaging platform	Specialty
Brian W. Pogue, PhD	Various systems	Various applications
Daniel A. Orringer, MD	Stimulated Raman histology	Neurosurgery
Cleopatra Charalampaki, MD, PhD	Multispectral imaging and confocal imaging	Neurosurgery

### 5.1 Summary of Important Points

• The uncoupling of tracers and imaging systems will allow for more streamlined approvals that separate drugs and devices from each other, where appropriate. This approach can provide quicker advancement and broadening of the field for other indications with new devices and drugs.
• Technical performance evaluation of fluorescence-guided surgery systems develops professional guidance on how to evaluate imaging systems and could eventually inform regulatory decisions.
• Three-dimensional (3D)-printed calibration phantoms can be used to quantify imaging system performance and allow investigators to accurately assess depth of penetration, concentration dependence, and spatial resolution.
• Stimulated Raman histology, a spectroscopic technology that uses individual Raman shifts of ${\mathrm{CH}}_{3}$ and ${\mathrm{CH}}_{2}$ bonds to generate contrast, offers a method of differentiating tumor from normal tissue in the operating room.
• Raman histology was able to provide a diagnosis from fresh tissue in <2.5  min. The results showed that this tool had an accuracy above 90% and could be a useful supplement to the information provided by neuropathologists.
• Multispectral imaging, which combines the blue signal of 5-ALA with the white light image, enhances surgeons’ ability to distinguish tumor tissue in real time.
• Confocal laser endomicroscopy (CLE) can provide real-time histological imaging with or without fluorescent contrast agents. CLE is capable of effectively distinguishing tumor margins *in vivo* under adequate conditions.

### 5.2 Review of Fluorescence Camera Imaging: Advantages Versus Disadvantages

Dr. Brian Pogue started the session by reviewing key criteria used in evaluating imaging systems, a timeline of important regulatory milestones, and some of the current opportunities and challenges in the field of fluorescent imaging technology. The talk started with an overview of regulatory milestones in the field that have advanced fluorescence-guided surgery systems in the past 20 years. He began with the 2004 approval of the Novadaq SPY (Stryker, Kalamazoo, Michigan) technology, which used x-ray angiography as part of its predicate for FDA clearance. This was the first time that fluorescence was approved to replace x-ray imaging, and it founded a new era in surgical guidance. Another major milestone occurred in 2017 when the FDA gave new drug approval to Gleolan as a precontrast agent for protoporphyrin IX, which was approved without prescribing a specific imaging device with it. The uncoupling of tracers and imaging systems will allow for more streamlined approvals that separate drugs and devices from each other, where appropriate. This approach can provide quicker advancement and broadening of the field for other indications with new devices and drugs.

Dr. Pogue touched on two specific developments within the field that are helping to realize this potential. The first is the working group for guidance for technical performance evaluation of fluorescence-guided surgery systems, which develops professional guidance on how to evaluate imaging systems and could eventually inform regulatory decisions. This group has developed a more extensive list of evaluation criteria across three categories: system performance tests, task-specific tests, and confounding effects.^38^ The second development, 3D-printed calibration phantoms, can be used to quantify imaging system performance in some of these categories by allowing investigators to accurately assess depth of penetration, concentration dependence, and spatial resolution. These advances, and others, will help researchers to continue improving upon current imaging systems and will aid surgical teams in determining which cameras are best suited to their needs and how to use systems in multicenter trials.^13^^,^^38^

### 5.3 Raman Spectroscopy Imaging of Brain Tumors on the Back Table

Dr. Daniel Orringer’s talk focused on potential back table imaging applications of stimulated Raman histology in neurosurgery and beyond. It is difficult but vitally important to distinguish tumor from healthy brain tissue. Stimulated Raman histology, a spectroscopic technology that uses individual Raman shifts of ${\mathrm{CH}}_{3}$ and ${\mathrm{CH}}_{2}$ bonds to generate contrast, offers a method of differentiating tumor from normal tissue in the OR. This label-free modality produces high-resolution images.^39^ Dr. Orringer talked about the first applications of this technology in animal models and used histological images of mouse brains to illustrate its capacity to depict structure on the neuronal level. Different levels of lipid density among cellular components yield clear contrast among nuclei, myelin, and other elements of brain tissue.^39^^,^^40^

After describing the basis of this technology, Dr. Orringer discussed some early human data and the translation of stimulated Raman histology to clinical use. Transitioning from the blue-green artificial coloring used in their early work to the pink purple of classic histological images made this technology more accessible to the broader clinical community. The development of a bedside system allowed this technology to be brought into the OR and nicely integrated into the surgical workflow. Specimens can be removed from the brain, gently compressed in an imaging chamber, placed in the bedside scanner, and visualized at high magnification and resolution within a matter of minutes. Features of these histological images such as cellularity can then be used to delineate tumor margins. In addition, Dr. Orringer’s group, to further investigate the potential of the technology, implemented a convolutional neural network to make predictions about the histological class of lesions based on the digital images. A training set was produced using 2.5 million images collected from 415 patients. The convolutional neural network was then tested on 278 patients across three institutions and was able to provide a diagnosis from fresh tissue in <2.5  min. The results showed that this tool had an accuracy above 90% and could be a useful supplement to the information provided by neuropathologists.^41^^,^^42^

### 5.4 Multispectral and Confocal Microscopy

The final presentation, given by Professor Cleopatra Charalampaki, covered confocal-assisted multifluorescent imaging in neurosurgery. At this time, 5-ALA is considered state-of-the-art in fluorescence-guided CNS tumor removal; however, there are some significant issues with this modality. Dark images, view obstruction due to bleeding, and the ergonomic limitations of switching between white light and fluorescent views reduce the utility of 5-ALA imaging in neurosurgery. Multispectral imaging, which combines the blue signal of 5-ALA with the white light image, enhances surgeons’ ability to distinguish tumor tissue in real time.^38^ Another advance that Dr. Charalampaki discussed was the use of multiple fluorophores, specifically 5-ALA and ICG, in a single procedure. The 5-ALA and ICG can be visualized through different channels on the same microscope, revealing tumor and adjacent vessels, respectively. Appropriate timing of tracer administration can also be used to illuminate the tumor with multiple fluorophores.^38^

Dr. Charalampaki then discussed CLE. CLE was first introduced in the field of gastroenterology in 2004 and can provide real-time histological imaging with or without fluorescent contrast agents. There are currently two FDA-approved CLE devices that are either integrated in or compatible with standard endoscopes. Dr. Charalampaki’s goals for introducing CLE in neurosurgery are to perform *in vivo* diagnosis, increase resection accuracy, and combine the technology with targeted tracers to perform light therapy.^38^^,^^43^^,^^44^

While promising, early applications of CLE with fluorescein revealed some challenges such as blood autofluorescence, poor depth of penetration, and narrow field of view. Transitioning to ICG limited blood interference and increased the depth of penetration, but obstacles remained. The need for direct contact with tissue makes CLE devices a class III product, increases regulatory burden, and limits specificity by tracer specificity. Despite these challenges, CLE is capable of effectively distinguishing tumor margins *in vivo* under adequate conditions.

Dr. Charalampaki ended by discussing the future directions of her and her team’s work. Their goals are to obviate the need for tissue contact and fluorescent labeling, expand the field of view, and increase the depth of penetration. Through a collaboration with Oxford University, development of a contactless, label-free CLE system has begun.^45^ This model, which uses pure infrared reflectance, has been used to successfully image animal and human tissue.

## 6 Conclusions

This biannual conference brought together leading research professionals versed in the latest clinical trials in IMI across a wide range of surgical specialties. While the majority of bench research focus in IMI currently lies on developing innovative dyes and imaging agents, this meeting spotlighted surgical investigators with practical experience in implementing IMI technology into patient care. Drawing on their past and current experience, several themes came forth during the event. One of the most important learning lessons from the conference was the variety of endpoints that were being established across different disease types.

**Fig. 2:**
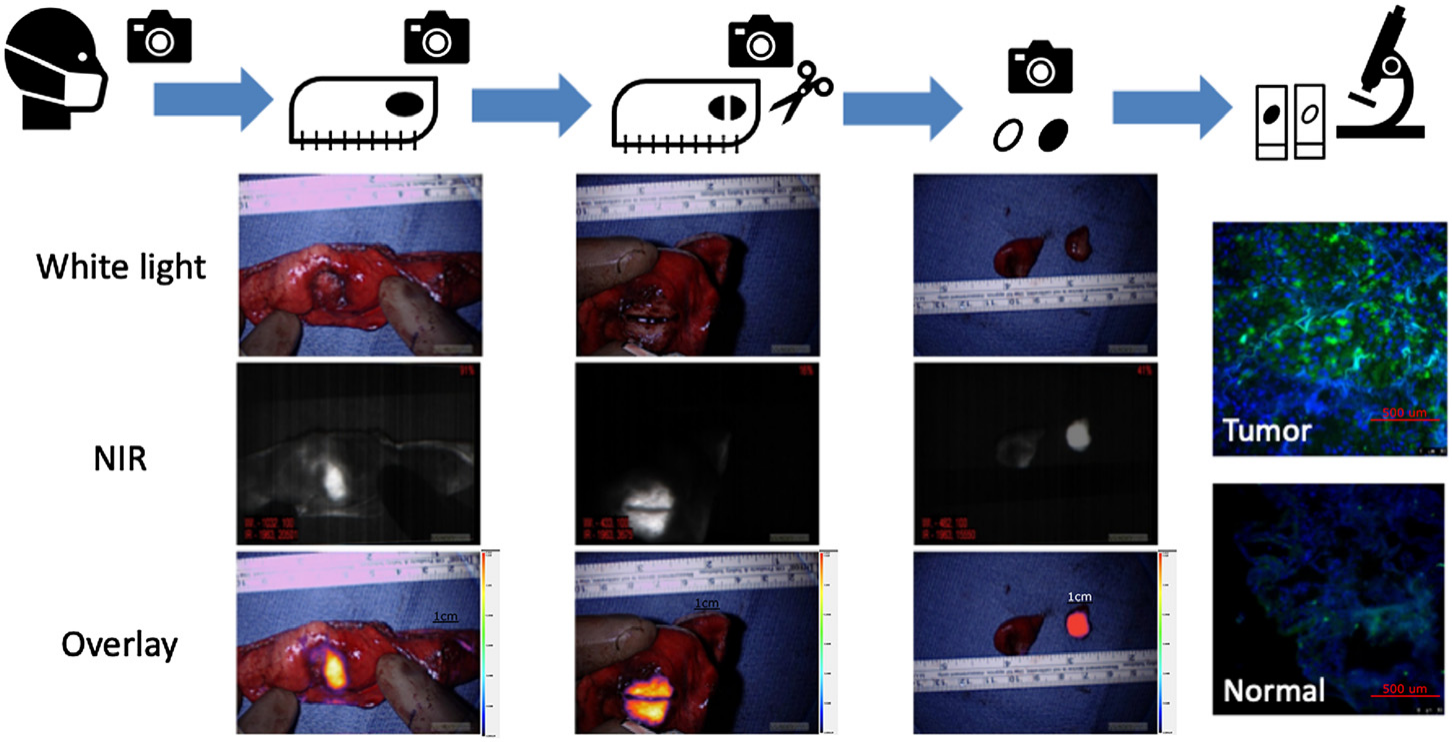
Utilization of IMI for various aspects of oncologic surgery ranging from lesion identification to optical biopsy.

The gold standard of result confirmation and oncologic success for surgical oncologists has for decades relied on sound histopathologic tissue assessment. This has remained the case for the past two centuries since the introduction of microscopy for pathological review in the 19th century.^46^ This paradigm places surgery as part of continuum that starts with preoperative evaluation (cross-sectional imaging, percutaneous tissue sample, etc.), followed by tissue resection and then completed with histologic analysis. IMI, on the other hand, plays a natural part in this continuum by bridging tissue resection and real-time tissue identification as shown in Fig. 2. This application of IMI, termed “optical biopsy,” was a common theme during the conference and served as an endpoint of trial success in various research presentations.^14^^,^^47^

For example, results from LUM015 studies (NCT03686215) in breast cancer presented by Dr. Smith demonstrated that IMI not only in real-time identifies diseased tissue but also can outperform standard histopathologic tissue analysis by identifying additional involved tissue that were otherwise missed by the pathologist. Similarly, anti-EGFR-based tracers can reliably assess the adequacy of oncologic nodule resection as well as inform the surgeon on the potential presence of synchronous lesions, lymph node involvement, and margin assessment in real time without interrupting flow of the operation. As acceptance of IMI and optical imaging becomes more commonplace in surgical oncology owing to the increase in large-scale, high quality clinical trials so will the natural utilization of the technology as an adjunct to the current gold standard histologic assessment. Nevertheless, identification of pathologic tissue remains a universally accepted goal for IMI in the scientific literature.

The theme of optical biopsy was also an integral part of the phase 3 trial evaluating the use of SGM-101 (anti-CEA antibody tracer) (NCT03659448) as described by Dr. Vahrmeijer. The tracer has the ability to distinguish malignant tissues from normal parenchyma in the liver, pelvis, omentum, and lymph nodes. Similar endpoints were assessed in trials that utilized NIR-II window for detection of hepatic lesions, which were very positive compared with conventional means. The phase 2 trial of OTL38 in lung adenocarcinomas (NCT02872701) presented by Dr. Singhal assessed the capacity of the tracer to distinguish malignant from benign tissue as a secondary endpoint with encouraging results, which is currently under further assessment in phase 3 trials. Naturally, in the era of molecular targeting, the concept of optical biopsy will be of immense value for the up-and-coming tracers.

As evident by the conference presentations, IMI has the potential to become an integral part of surgical management across a wide range of different specialties, cancer types, and organ systems. Although several specialties overlap in their surgical challenges, there are also unique issues in different cancer type resections. This theme was evident by the fact that the endpoints for utilizing IMI in different cancer type resections was varied. For example, the goal of OTL38 in the management of ovarian cancer (NCT03180307) is focused on appropriate intraperitoneal cytoreduction and tumor debulking, which would allow for the maximal benefit from adjuvant systemic therapy. However, in the management of lung adenocarcinomas (NCT02872701), OTL38 is primarily used for detection of synchronous lesions and margin verification as these variables are most prognostic of successful disease-free and overall survival (Fig. 1). The use of ICG-derived tracers is the most emblematic of this paradigm. Each specialty implements the same tracer to address different surgical needs, which differs depending on type of cancer involved and type of surgery performed. The ultimate utility of IMI is dependent on the technology successfully answering clinical challenges that are unique to each surgeon, organ system, and patient. A summary of tracers and their applications is shown in Table 6.

**Table 6:** Summary of tracers and their applications discussed during the seminar.

Tracer	Applications	Clinical challenge
5-ALA	Glioma, various neurosurgical applications	Margins
OTL38	Ovarian cancer	Cytoreduction
	Lung cancer	Synchronous lesions, margins
SGM-101	Colon cancer	Synchronous lesions, metastatic deposits, margins
LUM015	Breast cancer	Margins
Anti-EGFR antibody	Head and neck malignancies, sarcomas	Margins, synchronous lesions, lymph node involvement, metastasis
TumorGlow	Lung cancer	Localization
	Soft tissue sarcomas	Identification of synchronous lesions
	Pancreatic cancer	Margins
	Brain tumors	Margins
ONM-100	Prostate cancer	Margins, normal anatomic structure identification
ABY-029	Sarcomas	Margins
TumorPaint	Pediatric brain tumors	Margins
ALM-488	Normal nerve identification	Normal anatomic structure identification
Oxazine derivatives	Normal nerve identification	Normal anatomic structure identification

The outcome of successful oncologic resection for many solid tumors relies upon complete gross and microscopic removal of malignant tissues. Despite the majority of the focus being targeted toward primary lesion identified on preoperative imaging, morbidity and mortality of synchronous and metachronous lesions should not be ignored. Therefore, identifying these additional lesions that would be otherwise be missed remains a top priority, and this is where IMI plays an integral part in real-time surgical decision making. As demonstrated by clinical data in the phase 3 colon cancer trial (NCT03659448) in the Netherlands and advanced phase 2 lung adenocarcinoma trials (NCT02872701), detection of these lesions was of paramount importance. Similarly, these concerns were also echoed in phase 2 and 3 ovarian cancer trials (NCT03180307), where detection of lesions not identified by visual inspection was one of the endpoints for evaluating efficacy of IMI. The fact that these studies have found IMI helpful for intraoperative detection of additional disease at the time of surgery not previously identified on noninvasive imaging underscores the importance of IMI in oncologic surgery.^19^^,^^48^^,^^49^ For example, in the OTL phase 2 lung cancer trial, up to 12% of the patients had additional cancers that were not seen on preoperative CT scans.

Another example of variations in specialties is that the importance of identifying additional lesions is not as paramount in brain cancer surgery as it is in the management of solid tumors in the pelvic or thoracic cavity. As pointed out by Hadjipanayis et al. and Lee et al., in HGG surgery, identifying of synchronous and metachronous lesions is accomplished by high spatial fidelity of MRI, which has been validated to detect brain lesions down to 1 mm. Nevertheless, the advantage of IMI in neurosurgical oncological procedures lies in its ability to assess the margin status in the tumor bed. In fact, 5-ALA has been shown to be >80% effective in evaluating margins in HGG, and this is significant given that R0 resection is the primary determinant of disease-free and overall survival in HGG.^13^ Use of IMI in margin assessment has been taking an ever more important role over the last few years as it has been found to be superior to conventional methods in breast surgery, head and neck cancers, and sarcoma resections in early pilot and phase 2 studies. This was best exemplified by encouraging and practically sound data from the anti-EGFR antibody-based tracers in head and neck malignancies and axial sarcoma resections. Similar positive results were noted using cathepsin-activated enzymatic tracers in breast cancer resections, which serve as a steppingstone for multinational phase 3 studies (NCT03686215). Unlike the previous meetings, this year there was a dedicated session on back table assessment of margins and use of IMI in tissue confirmation such as Raman spectroscopy. The technology is naturally branching out from its real-time intraoperative use to other aspects of surgical oncology.

While the use of IMI has important implications in identifying malignant tissues for sound oncologic resection, its ability to detect normal anatomical structures should not be overlooked. Surgical oncologists often operate in areas with multiple functionally sensitive motor and sensory nerves. Damage to these structures can result in severe functional limitations and increase postoperative morbidity. These are particularly relevant for head and neck malignancies, orthopedic malignancies, and pelvic surgical dissections. Development of new tracers allows for implementation of IMI in identifying these key structures that are often not visible to the naked eye or are hidden behind tumor where the dissection is taking place. Data from preliminary and phase 1 studies looking at ALM-488 (NCT04420689) and oxazine-derived dyes have shown that normal neural structures can fluoresce and provide real-time data for surgical decision making. In unison, the ability of IMI to detect cancerous lesions and assessment of microscopic disease at the tumor in combination with normal structure identification makes it a viable tool in the surgeons’ armamentarium.

In summary, IMI is increasingly being recognized as an integral component of oncologic surgical resections and will likely change the face of cancer surgery for many years to come. Since the FDA approval of 5-ALA for HGG, the field has been progressing at a rapid rate and is pushing the boundaries of IMI beyond conventional intraoperative imaging. There are common challenges encountered among cancer surgeons across multiple specialties, and the next decade will surely show the value of IMI in tackling those problems.
